# Using screening scores to provide feedback to patients on their alcohol-related risks: the association between AUDIT-C scores and average consumption and health outcomes

**DOI:** 10.1186/1940-0640-8-S1-A13

**Published:** 2013-09-04

**Authors:** Katharine A Bradley, Anna D Rubinsky, Emily C Williams, Gwen T Lapham, Carol Achtmeyer, Daniel R Kivlahan

**Affiliations:** 1 Group Health Research Institute, Seattle, WA, USA; 2 Health Services Research & Development (HSR&D) Northwest Center of Excellence, Veterans Affairs (VA) Puget Sound Health Care System, Seattle, WA, USA; 3 Center of Excellence in Substance Abuse Treatment and Education (CESATE), Veterans Affairs (VA) Puget Sound Health Care System, Seattle, WA, USA; 4 Department of Medicine, University of Washington, Seattle, WA, USA; 5 Department of Health Services, University of Washington, Seattle, WA, USA; 6 Center for Health Care Evaluation, Veterans Affairs (VA) Palo Alto Health Care System, Palo Alto, CA, USA; 7 General Medicine Service, Veterans Affairs (VA) Puget Sound Health Care System, Seattle, WA, USA; 8 Department of Psychiatry and Behavioral Sciences, University of Washington, Seattle, WA, USA

## Introduction

This study integrates two lines of research on the AUDIT-C: a study which estimated average alcohol consumption at each AUDIT-C score using national U.S. population data, and a set of studies which evaluated the association of AUDIT-C scores with alcohol-related health outcomes. This presentation synthesizes results from these studies to depict the association between 1) AUDIT-C scores, and 2) mean daily alcohol consumption as well as adverse alcohol-related health outcomes.

## Methods

Mean daily alcohol consumption (drinks/day) at each AUDIT-C score (0-12 points) was evaluated in U.S. adults who provided detailed reports of consumption on the 2000-2001 National Epidemiologic Survey on Alcohol and Related Conditions (NESARC) and reported past-year drinking (n 26,576). Analyses were limited to NESARC participants age 45 and over to parallel the age of the samples in the health outcomes studies (below). The associations between AUDIT-C scores and subsequent risk for the following alcohol-related health outcomes were assessed in U.S. Veterans Health Administration (VA) patients: patient self-management of hypertension and diabetes; medication adherence; surgical complications; hospitalizations for upper GI bleeding, liver disease, and pancreatitis; trauma; inpatient health care utilization, and mortality.

## Results

The presentation will graphically depict the relationship between AUDIT-C scores and mean daily alcohol consumption (as AUDIT-C scores increase, mean daily alcohol consumption increases from 0 to17 drinks/day) and identify AUDIT-C scores and mean daily alcohol consumption associated with each adverse health outcome. For example, patients with AUDIT-C scores of 7 drink an average of 2.7 drinks daily and are at increased risk for medication non-adherence, postoperative complications, and GI hospitalizations.

## Conclusions

This information may help primary care clinicians recognize the value of a scaled alcohol screen, routinely used in some primary care settings, including its utility for providing patients with feedback on their alcohol-related risks during brief alcohol interventions.

